# Letter in response to: “The importance of clinical importance when determining the target difference in sample size calculations”

**DOI:** 10.1186/s13063-023-07695-1

**Published:** 2023-10-12

**Authors:** Hubert Wong

**Affiliations:** School of Population & Public Health, University of British Columbia, 2206 East Mall, Vancouver, BC V6T 1Z3 Canada

To the Editors:

I thank Parker and Cook for presenting their position on the role of the clinically important difference or minimum important difference (MID), when setting the target difference (what I call the assumed benefit—I will use the two terms interchangeably) in superiority trial sample size calculations [1]. It is an issue which I believe continues to be misunderstood widely in practice and deserves further dialog. Indeed, my Commentary [2] which motivated theirs was not intended to dismiss the relevance of MID in deciding whether a trial should proceed but to point out the potential consequences of setting the MID as the target difference for *calculating* the required sample size. I share nearly all the sentiments that Parker and Cook have expressed with respect to the challenges in sample size calculation and the practical considerations, so it is curious how far apart our positions appear to be (though perhaps are less so in truth). Part of the explanation is that they have misconstrued the message from my Commentary, so I will address that issue first. But in addition, I will elaborate on what appears to be a difference in our beliefs about what is achieved in a trial in which the target difference has been set to the MID and conclude by suggesting that a Bayesian treatment could resolve the discordance.

The *true* power of a trial is determined by design inputs (randomization scheme, sample size) and decision criteria (analysis model, type I error rate, etc.) which are chosen by the trial designer, in combination with parameters whose values are unknown and beyond the control of the trial designer (population characteristics, including the *true* benefit of the intervention). We can never know the true power as it depends on the unknown parameter values. Instead, we perform calculations for a *target* power using *assumed* values for these unknown parameters—for simplicity in the discussion, suppose the only one unknown is the true benefit. My point was that in order for the true power and the target power to match, the assumed benefit must match the true benefit. When this holds, the term “valid” seems an appropriate descriptor for that calculation; some might prefer to use “accurate” or “correct,” but I deliberately avoided those choices because they could be misinterpreted as referring to only technical correctness of the calculation, rather than the calculation providing a result that *reflects truth about what the trial will accomplish in the real world*. Other choices for the assumed benefit are not “valid” in that using those values will result in a trial whose true power differs from the target power. No other connotation (e.g., validity of the trial, or the analysis) was implied, and I will not address comments Parker and Cook made stemming from those connotations.

Parker and Cook write “The goal of a sample size calculation is not to have “the ‘target power’ that ‘matches’ the ‘true power’, …”. Indeed, this is not the motivating purpose of a sample size calculation, but it is a condition that we must believe to be satisfied (approximately) for the sample size calculation to have any worth—if it does not hold, the calculation *misleads* us about the true power, so we should never accept calculations in which we doubt that the assumed benefit is realistic. The fact that we do not know the true benefit does not absolve us from making this judgment. However, what constitutes a clinically important difference has no impact on what is the true benefit and hence whether a given sample size is valid. I emphasize that I am not implying that believing one has a valid sample size calculation is an *endorsement* for the trial to be conducted, as Parker and Cook incorrectly inferred in their example where the true benefit and realistic difference happen to coincide but is less than the clinically relevant difference. As pointed out in my Commentary, irrespective of how the realistic benefit is set, the MID plays a role in assessing whether it would be worthwhile to pursue the trial, and in their example my approach would argue that the trial would not be justified because it is unlikely the trial will find a clinically important benefit and the trial should not be conducted, in line with Parker and Cook’s conclusion.

The difference between our views is that I believe one should separate out considerations of realistic benefits, which aims to assess how much power we expect the trial will achieve (a mathematical criterion), from considerations of the MID, which should be used to help assess whether the inferences from the trial are likely to be useful for clinical decision-making (a value criterion). Parker and Cook acknowledge the separation, but it is unclear to me how they are reconciling this difference. Simplistic rules that mix up these two aspects can lead us astray. As a real example, consider recent work on the use of psilocybin for treatment of depressive illnesses. Much of the preliminary data (with and without comparator groups) have suggested a magnitude of benefit that is much greater than what most people would consider a minimally important benefit. For a recent grant application, we proposed a trial proposal assuming a “conservative best guess” of the true benefit of psilocybin for the target difference, which resulted in a feasible sample size. If we had assumed instead a smaller value reflecting the MID, the sample size would have more than doubled, making the trial infeasible. That is, using the MID would result in not doing a trial whose conduct in fact is supported strongly by the evidence and which could be highly impactful on care. If one argues that there was no need to reduce the target difference to the MID, since the conservative best guess was already both “realistic” and “clinically important,” then that simply emphasizes the point that the MID is not relevant to the calculation! Now consider the converse where the most likely values of the benefit are less than the MID. For example, suppose it has been decided that the MID is 5 units and that current evidence suggests the best estimate for the true benefit is 3 units, and a benefit as large as 5 units is still plausible, albeit less likely. It could be tempting to conclude that it would be appropriate to set the target difference to 5 units since it is both plausible and the minimal clinically important value. But clearly, the *expectation* is that the true effect is somewhat lower, so our assessment ought to be that using MID likely means the trial will be underpowered, and again it is not a good idea to adopt the MID. Finally, consider a scenario where the MID is 5 and lies within the range of realistic estimates for the true benefit, 3 to 10, say. If one argues that we should choose a value towards the lower end to ensure we are not underpowered, but we should not consider differences that are not clinically important, then one might conclude the target difference should be set to the MID of 5. Proceeding in this way could be reasonable if it meant that there was a high probability that at the end of the trial, we could actually conclude with confidence whether the true benefit is or is not greater than the MID. But we know this is not so.

Parker and Cook argue that in fact trialists do not really expect to obtain such a definitive conclusion and only expect to have “sufficient sample size to reach a conclusion of statistical significance and estimate the treatment effect with adequate precision.” But if that is all that is expected, what is the rationale for setting the target difference specifically to the MID? Under standard assumptions (i.e., two-sample *t*-test, two-sided alpha = 0.05, power = 0.8), the standard error will be 0.35 times the MID. What makes this standard error “adequately precise,” and why should this standard error be preferred over what one would get if the target difference had been set to 0.8 times the MID, or 1.5 times the MID, or some value not necessarily derived from the MID? Without better justification, using the MID strikes me as not much more than a convenient convention that attains a somewhat arbitrary evidentiary standard.

I will not debate what fraction of trialists do not expect to obtain a definitive conclusion, but in my experience, many are naive about what a trial can actually demonstrate. That is, I think many do believe that the trial will have high power to show whether a clinically meaningful benefit exists—and they are not at fault for having this misunderstanding. Consider how conversations about sample size often begin with the trialist being asked “How large a benefit would be needed to impact clinical practice?” and how they are guided to “pitch” their trial as capable of changing practice! This sometimes leads to awkward conversations where one has to explain how a trial that was designed to “detect a clinically important difference of 5 units” and for which the trial result was “positive” (ended up with a 95% confidence interval of (0.1, 7.1), say) actually indicates that it is unlikely that a 5 unit benefit exists! Such discordances arise because we attempt to “shoe-horn” the multiple relevant considerations into calculations within the conventional framework, when in fact the framework is too simplistic to allow for proper accounting of these considerations. Parker and Cook noted that Bayesian approaches (see Kuzmann et al. [3] for a recent review) to sample size calculation have been developed. While the specifics of these approaches are outside the scope of the current discussion, it is important to recognize that these approaches typically do allow one to incorporate uncertainty about the true benefit and to treat the clinically important difference as a distinct input which serves a separate role. I would be interested in hearing Parker and Cook’s position on whether there would be value in switching to such a framework.

## Data Availability

Not applicable.
